# *R*2-Based Multi/Many-Objective Particle Swarm Optimization

**DOI:** 10.1155/2016/1898527

**Published:** 2016-08-28

**Authors:** Alan Díaz-Manríquez, Gregorio Toscano, Jose Hugo Barron-Zambrano, Edgar Tello-Leal

**Affiliations:** ^1^Facultad de Ingeniería y Ciencias, Universidad Autónoma de Tamaulipas, 87000 Victoria, TAMPS, Mexico; ^2^Cinvestav Tamaulipas, Km. 5.5 Carretera Ciudad Victoria-Soto La Marina, 87130 Victoria, TAMPS, Mexico

## Abstract

We propose to couple the *R*2 performance measure and Particle Swarm Optimization in order to handle multi/many-objective problems. Our proposal shows that through a well-designed interaction process we could maintain the metaheuristic almost inalterable and through the *R*2 performance measure we did not use neither an external archive nor Pareto dominance to guide the search. The proposed approach is validated using several test problems and performance measures commonly adopted in the specialized literature. Results indicate that the proposed algorithm produces results that are competitive with respect to those obtained by four well-known MOEAs. Additionally, we validate our proposal in many-objective optimization problems. In these problems, our approach showed its main strength, since it could outperform another well-known indicator-based MOEA.

## 1. Introduction

Evolutionary Algorithms (EAs) encompass a set of bioinspired techniques that make a multidimensional search and have been found to be effective in locating solutions close to the global optimum even in highly rugged search spaces. EAs are suitable alternatives for solving problems with two or more objectives (the so-called multiobjective optimization problems or MOPs for short), since they are able to simultaneously explore different regions of the search space and obtain several points from the trade-off surface in a single run. Since the mid-1980s, the field of evolutionary multiobjective optimization (EMO) has grown and a wide variety of multiobjective EAs (MOEAs) to solve real applications have been proposed so far.

Moreover, the use of Pareto dominance (PD) to solve multiobjective optimization problems (MOPs) has been successfully used for several years. However, when the number of objectives increases, the proportion of nondominated solutions also increases but in an exponential way [1–3]. Therefore, very quickly, it becomes impossible to distinguish individuals for selection purposes. Such a behavior dilutes the selection pressure, since the choice of solutions is performed practically at a random way.

For this reason, the evolutionary multiobjective optimization (EMO) community has developed several approaches to overcome this shortcoming in the fitness assignment process [4, 5]. Several of these approaches drive the search using a quality assessment indicator. This idea has become more popular in the last few years, mainly because of the growing interest in tackling multiobjective problems having 4 or more objectives (commonly called “many-objective optimization problems” or MaMOP for short), for which indicator-based MOEAs seem to be particularly suitable [6]. The idea of using an indicator-based selection is to identify the solutions that contribute the most to the improvement of the performance indicator adopted in the selection mechanism [7, 8].

The Indicator-Based Evolutionary Algorithm (IBEA) proposed by Zitzler and Künzli [9] is the most general version of an algorithm of this sort. Instead of using the problem at hand as a fitness function, these methods minimize or maximize (whichever the case) a performance indicator. Originally, IBEA was proposed to be used with two different performance measures: hypervolume [10] and the *ϵ*-indicator. Among other results, Zitzler and Künzli found that no additional diversity preservation mechanism was required when ranking the solutions with an indicator. Similarly, Beume et al. [11] proposed *S* Metric Selection Evolutionary Multiobjective Algorithm (SMS-EMOA). However, in this case, Beume et al. replaced the Crowding Distance with the hypervolume indicator. Ishibuchi et al. [12] presented a novel approach that iteratively optimizes separately each objective and searches for the solution that contributes more to the hypervolume indicator. This approach was designed to search for a small number of nondominated solutions along the entire Pareto front. Igel et al. [13] proposed the Multiobjective Covariance Matrix Adaptation Evolution Strategy (MO-CMA-ES). This algorithm uses a set of monoobjective optimizers as its population. Each optimizer generates new solutions that can be accepted back into the population according to their ranking with respect to PD or their contribution to the hypervolume.

Although the hypervolume's nice theoretical property has positioned it as the most popular choice for implementing indicator-based MOEAs [11], it is well-known that its computational cost considerably increases as we raise the number of objectives. To overcome this drawback, some researchers [6] have opted for approximating the hypervolume. However, this sort of scheme can decrease the accuracy of the selection mechanism. Rodríguez Villalobos and Coello Coello [14] recently proposed Δ_*p*_-differential evolution in which the authors adopted Δ_*p*_ performance indicator [15] as an alternative to the hypervolume. The fitness assignment for each solution is performed through the contribution to Δ_*p*_. Δ_*p*_ indicator requires a reference set to be calculated, and the authors used the nadir point and the ideal vector to create such a reference set. The authors reported that this approach could obtain competitive results with respect to other MOEAs (including SMS-EMOA), having as its main advantage its very low computational cost, even when dealing with many-objective problems.

Moreover, the PSO has been used to solve a lot of problems [16, 17]. However, in the literature the works with respect to the use of an indicator to guide the search of a PSO are very limited. In [18], Padhye uses the contribution to the hypervolume to select the *gbest* and the *pbest* of the PSO. However, in this work the hypervolume is not used to select the particles that advance to the next iteration. This algorithm is used for the topology optimization of a compliant mechanism. In [19], the authors proposed hybridization of MOPSO with a local search operator. The proposed MOPSO uses an indicator to truncate an external archive of solutions. In this work, two approaches were proposed one that uses the *ϵ*-indicator and another that adopts the hypervolume performance measure. Both approaches reached similar results. However, these approaches were compared only with problems with 2 and 3 objectives.

Other suitable performance indicators are the *R*2 indicator [20]. In recent works, it has been reported that desirable properties (i.e., it is weakly monotonic, it produces well-distributed solutions, and it can be computed in a fast manner) make the *R*2 indicator a viable candidate to be incorporated into an indicator-based MOEA [7, 21–25]. In such works, the *R*2 behavior has been compared with respect to that of the hypervolume and concludes that they both have a similar behavior, but *R*2 has a considerably lower computational cost.

In [23], the authors proposed an approach to fast ranking the population (of a genetic algorithm and a differential evolution) using the *R*2 indicator. Although their approach is able to work with many-objective problems, it uses an external archive. Therefore, the metaheuristic at hand has to be highly modified in order to work with the approach. In this paper, we propose a *R*2-based multiobjective approach that maintains the nature of PSO, while empowering it to handle many-objective problems.

In this work, we propose to use the *R*2 indicator to guide the search of MOPSO. The new approach is then compared with respect to some state-of-the-art MOEAs taken from the specialized literature. Furthermore, we present a scalability study to analyze the behavior of our proposed approach as the number of objectives of the problem increases. The remainder of this work is organized as follows. Details of the *R*2 indicator are given in Section 2.  Section 3 presents our proposed approach. A comparative study with respect to other algorithms is presented in Section 4. Finally, Conclusions and Future Work are given in Section 5.

## 2. *R*2 Indicator

The family of *R* indicators [20] is based on utility functions which map a vector $\vec{y}\in {ℛ}^{k}$ to a scalar value *u* ∈ *ℛ* in order to measure the quality of two approximations of the Pareto front.

**Definition 1 .**

For a set *U* of utility functions, probability distribution *p* on *U*, and a reference set *R*, the *R*2 indicator of a solution set *A* is defined as

(1) R2R,A,U,p=∫u∈Umaxr∈R⁡urpudu−∫u∈Umaxa∈Auapudu.

**Definition 2 .**

For a discrete and finite set *U* and uniform distribution *p* over *U*, the *R*2 indicator can be defined as [26]

(2) $$\begin{array}{c} R2\left(\;R,A,U\;\right)=\frac{1}{\left|U\right|}\underset{u\in U}{\sum }\left(\;\underset{r\in R}{\max}\left\{\;u\left(\;r\;\right)\;\right\}-\underset{a\in A}{\max}\left\{\;u\left(\;a\;\right)\;\right\}\;\right). \end{array}$$

Since the first summand (max_*r*∈*R*_{*u*(*r*)}) is constant if we assume a constant *R*, the first summand can be deleted in order to have unary indicator as a result (called *R* for simplicity) [21].

**Definition 3 .**

For a constant reference set, the *R*2 indicator can be defined as unary indicator as follows:

(3) $$\begin{array}{c} R2\left(\;A,U\;\right)=-\frac{1}{\left|U\right|}\underset{u\in U}{\sum }\left\{\;u\left(\;a\;\right)\;\right\}. \end{array}$$

We selected the Tchebycheff function to be used as the utility function of our approach. This function works well when optimizing different types of Pareto fronts. Also, this aggregation function is not smooth for continuous multiobjective problems. However, our algorithm does not need to compute the derivative of the aggregation function. The Tchebycheff function can be defined as $u(z)=u_{\lambda }(\vec{z})=-{max}_{j\in \{1,\ldots ,k\}}\lambda_{j}|z_{j}^{*}-z_{j}|$, where *λ* = (*λ*_1_,…, *λ*_*k*_) ∈ Λ is a weight vector and *z* ^*∗*^ is utopian point (an objective vector that is not dominated by any feasible search point).

**Definition 4 .**

The *R*2 indicator of a solution set *A* for a given set of weight vectors Λ and utopian point *z* ^*∗*^ is defined as

(4) $$\begin{array}{c} R2\left(\;A,\Lambda ,z^{*}\;\right)=\frac{1}{\Lambda }\underset{\lambda \in \Lambda }{\sum }\underset{a\in A}{min}\left\{\;\underset{j\in \left\{1,\ldots ,k\right\}}{max}\left\{\;\lambda_{j}\left|\;z_{j}^{*}-a_{j}\;\right|\;\right\}\;\right\}. \end{array}$$

**Definition 5 .**

Finally, we say that the contribution of one solution *a* ∈ *A* to the *R*2 indicator can be defined as

(5) CR2a,A,Λ,z∗=R2A,Λ,z∗−R2A∖a,Λ,z∗.

## 3. Proposed Approach

### 3.1. The PSO Algorithm

PSO has been successfully used for both continuous nonlinear and discrete binary single objective optimization [27]. The pseudocode of PSO is shown in Algorithm 1.

### 3.2. PSO Based on the *R*2 Indicator (*R*2-MOPSO)

PSO has been particularly suitable for multiobjective optimization mainly because of the high speed of convergence that the algorithm presents. Based on such behavior, one would expect a multiobjective PSO (MOPSO) based on indicators to be very efficient computationally speaking. However, it is necessary to perform two main modifications to the original PSO:

- To modify the algorithm to handle multiple objectives and produce a set of nondominated solutions in a single run.
- To modify the algorithm to obtain good distribution of solutions.

A natural modification to a PSO algorithm aimed to handle multiple objectives which is to replace the comparison operator in order to determine whether a solution *a* is better than a solution *b*. Most of the existing approaches use the Pareto ranking scheme to extend the PSO to handle multiobjective optimization problems. However, with a Pareto ranking scheme a set of nondominated solutions will be produced (by definition, all nondominated solutions are equally good). Having several nondominated solutions implies the inclusion into the algorithm of both additional criteria to decide whether a new nondominated solution is *pbest* or *gbest* and a strategy to select the guide particles (*pbest* and *gbest*). Therefore, in our proposal the contribution to the *R*2 indicator (see (5)) is used to replace the comparison operator. Additionally, an external file *A* of nondominated solutions is used with all the evaluated solutions found in the optimization process. If the number of nondominated solutions is greater than the limit of the file, we select the solutions that represent the best *R*2 contributions. The utopian point (*z* ^*∗*^) is formed with respect to the best obtained values for each objective (of this external file). This utopian point is updated when the external file changes.

The MOPSO implemented in this paper is called the *R*2-MOPSO. At the start of the optimization cycle, all the *N* particle positions are initialized randomly and their velocities are set to zero. At the onset *pbest* for each particle is assigned as the particle itself. A different *gbest* is obtained for each particle. As *gbest*, *pbest* was selected with the best contribution to the *R*2 indicator. Next, the velocity and position of the particle are updated. Afterwards, the new position of the particle is evaluated, and the contribution to *R*2 is calculated for the union of all the *pbest* positions with the new position of the particle, so that if the new position has a better *R*2 contribution than its *pbest* then the *pbest* of the particle will be replaced with the new position. Moreover, if the new position has a similar *R*2 contribution than its *pbest* then either one is chosen randomly to be the new *pbest*.

An important feature of *R*2-MOPSO is that the Pareto dominance is completely removed from the evolutionary process. The Pareto dominance is only used in the external file. However, this external file is not used in the optimization process of the PSO. Thus, we can say that *R*2-MOPSO maintains the essence of the original PSO. We show the pseudocode of our *R*2-MOPSO in Algorithm 2.

### 3.3. Weight Vectors

In order to compute the *R*2 indicator, it is necessary to have a set of uniformly distributed weight vectors in order to obtain good distribution of solutions. For biobjective problems and for three-objective problems, we compute each weight vector as follows:

(6) $$\begin{array}{c} \left(\;\epsilon ,\frac{1}{H},\frac{2}{H},\ldots ,\frac{H}{H}\;\right), \end{array}$$

where *H* controls the number of weight vectors and *ϵ* is a value close to zero (*ϵ* = 10^4^ is suggested), in order to prevent cancellation in the calculations. The total number of vectors is represented by *C*_*m*−1_^*H*+*m*−1^. In this work, for biobjective problems, we decided to use *H* = 99, and for three-objective problems we adopted *H* = 23.

However,  for many-objective problems the weight vectors were randomly initialized in such a way that the sum of each weight vector is equal to one (the random weights' vector was generated as in MOMHLib++ [28]).

## 4. Performance Assessment

The proposed approach was evaluated using 15 test functions. Five functions were taken from the Zitzler-Deb-Thiele (ZDT) test suite [29], six test problems were taken from [30], and the remaining were taken from the Deb-Thiele-Laumanns-Zitzler (DTLZ) test suite [31]. The main features of these test problems are shown in Table 1.

In order to assess the performance of the proposed approach, we decided to include the hypervolume performance measure.

(*1) Hypervolume (HV*). The HV computes the area covered for all the solutions in the approximated Pareto front *Q* with the help of a reference point *W*. Equation (7) shows the mathematical definition of HV:

(7) $$\begin{array}{c} HV=volume\left(\;\overset{\left|Q\right|}{\underset{i=1}{⋃}}v_{i}\;\right), \end{array}$$

where, for each solution *i* ∈ *Q*, a hypercube *v*_*i*_ is constructed using the reference point *W*. Therefore, HV is the union of the volume of all the hypercubes.

### 4.1. Experiment 1 (Two and Three Objectives)

In order to make a comparative study, we chose the four following approaches: (1) NSGA-II (this is, by far, the most popular Pareto-based MOEA), (2) MOEA/D (a more recent MOEA, based on decomposition, which has been found to be more effective than NSGA-II in a number of problems), (3) SMS-EMOA (this is perhaps the most popular indicator-based MOEA in use today), and (4) MOMBI-II (an indicator-based MOEA based on the *R*2 indicator). All these MOEAs adopted a population size of 100 (except for MOEA/D in problems with 3 objectives where the population size was of 105), and the remainder parameters for each algorithm were the ones suggested by their authors.  Table 2 summarizes the parameters adopted in our comparative study.

Since we would like to investigate about behavior of the algorithms, we decided to measure the online convergence. Therefore, 30 independent executions were performed, and we measured the HV every 100 evaluations during 20,000 function evaluations in biobjective problems and 30,000 function evaluations in problems with three objectives.

**Figure 1:**
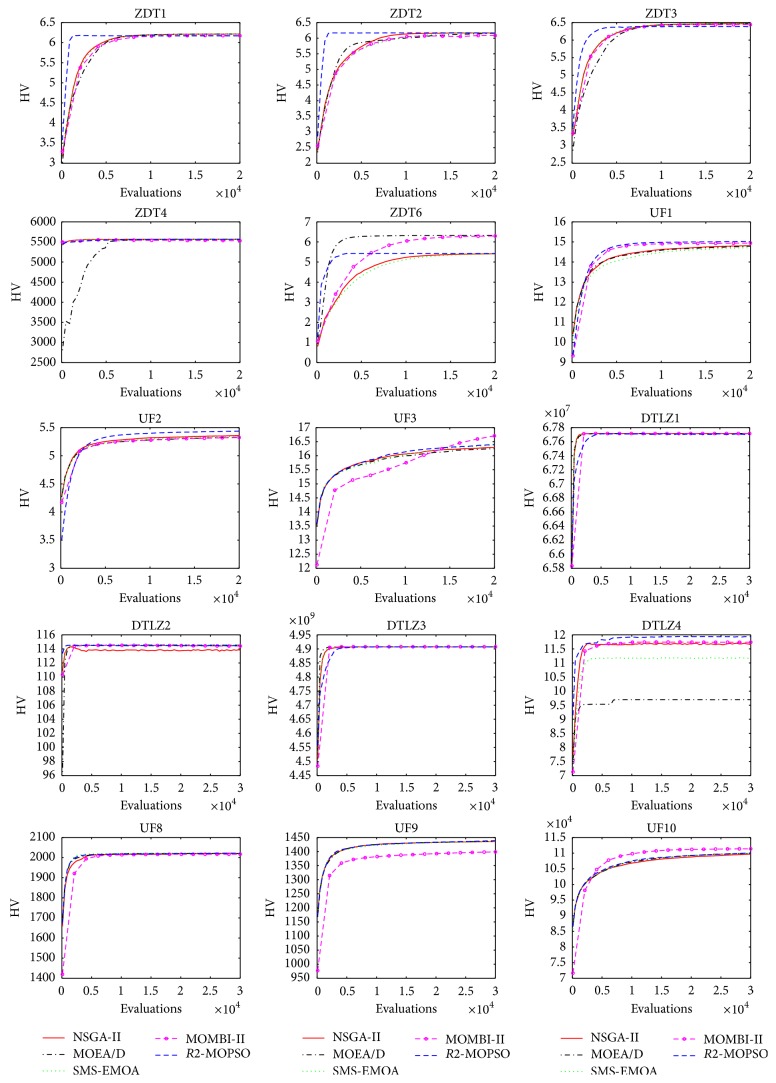
Graphical results of the online convergence for all the test problems.

Figure 1 shows the results of the MOEAs in all the adopted problems. In this figures, *x*-axis shows the number of evaluations and *y*-axis shows the average performance according to the HV. A better performance in most of the problems with two objectives (ZDT1, ZDT2, ZDT3, and ZDT6 and UF1 and UF2) of *R*2-MOPSO with respect to the other algorithms can be observed. A rapid convergence in this type of problem is shown. However, in ZDT4 the *R*2-MOPSO produced a slower convergence than the other approaches. Moreover, in three-objective problems the behavior of the *R*2-MOPSO is similar than the rest of the algorithms.

The application of the hypervolume performance measure to the results obtained by the four approaches is shown in Table 3. From these results, it is easy to see that all the approaches behaved similarly. The NSGA-II slightly outperformed others for the ZDT test problems. However, this approach did not behave well when optimizing the three-objective test problems. It can be observed that the *R*2-MOPSO are competitive in most of the problems with respect to the results obtained by the other algorithms.

**Figure 2:**
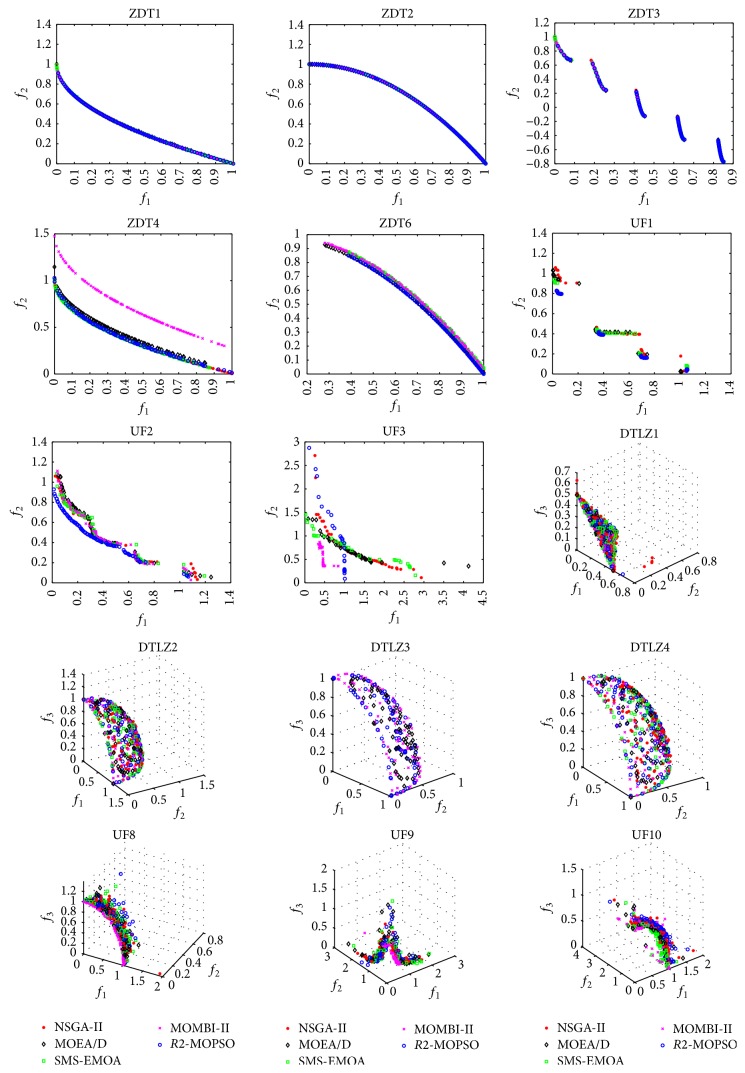
Graphical results of the visual comparison (Pareto fronts) for all the test problems.

Additionally, a visual comparison was performed in order to help to understand the obtained results. In this experiment the median of the executions with respect to the hypervolume indicator was plotted. In Figure 2 the produced Pareto fronts are shown. From this figure, it can be noticed that the *R*2-MOPSO is competitive with respect to the state-of-the-art algorithms in most of the problems.

### 4.2. Experiment 2 (Statistical Analysis)

Additionally, a statistical analysis was performed in order to verify the statistical differences of the proposed approach with respect to the SMS-EMOA and MOMBI-II. The comparison methodology is as follows: first, the Shapiro-Wilk test was performed to verify the normality of the data distributions. If both samples are normally distributed, the homogeneity of their variances is verified with Bartlett's test. If both variances are homogeneous then an ANOVA test is performed to verify the statistical significance; otherwise Welch's test is used. For nonnormally distributed data, the Kruskal-Wallis test was performed to verify the statistical significance. In all the test a significance level of 0.05 was used. The results are presented in Table 4. The values marked with “+” suggest a statistically significant difference in performance in favor of our proposal, while the values marked with “−” suggest a difference in favor of the state-of-the-art algorithms. Additionally, the values in blank suggest a similar performance. The obtained results confirm the above discussion. The proposed approach showed a similar statistical performance with respect to the MOMBI-II. However, in some problems (UF1, UF2, DTLZ1, DTLZ3, and DTLZ4), a statistically significant difference in favor of the proposed approach with respect to the SMS-EMOA can be observed.

### 4.3. Experiment 3 (Many-Objective Problems)

Since one of the aims of using an indicator-based MOEA is its capability to perform well in the presence of many objective functions, we decided to test the behavior of the *R*2-MOPSO in such problems. For this experiment, we focused our efforts on solving the DTLZ{1–4} test problems in order to investigate the behavior of our proposed approach as the number of objectives of the problem increases (we increased from 4 to 10 objectives). Our results are compared with respect to those obtained by SMS-EMOA and MOMBI-II. Each MOEA was executed 30 times and their results were evaluated using the hypervolume indicator. The reference points used by the adopted problems were of [1.5] for DTLZ1 and [2.0] for the remaining problems. The parameters were similar to those adopted in the previous experiment. Since the computational time required by the original SMS-EMOA algorithm (using exact hypervolume) becomes prohibitive very quickly, the number of objectives is raised (this has also been illustrated in other works [14]). For this reason, we decided to compare our approach with a modified version of SMS-EMOA. This version uses the approximation to the contribution to the hypervolume (proposed in [6]) in order to decrease the execution time of the algorithm. The number of samples used in this latter algorithm is 100,000.

Additionally, the same statistical analysis performed in experiment two was realized in this experiment. The values marked with “(+)” suggest a statistically significant difference in performance in favor of our proposal, while the values marked with “(−)” suggest a difference in favor of the state-of-the-art algorithms. Moreover, the values in with “( )” suggest a similar performance.

The results for the compared approaches are shown in Tables 5, 6, 7, and 8. In some cases, the hypervolume has a value of zero. This value indicates that the algorithm did not not achieve any nondominated solution with respect to the reference point. For DTLZ2 and DTLZ4 shown in Tables 6 and 8, respectively, the results of the compared approaches were similar (these two problems were easiest to solve in our benchmark). However, for DTLZ2 from 7 objectives, the *R*2-MOPSO is slightly better than SMS-EMOA, and in DTLZ4 the *R*2-MOPSO is always better than SMS-EMOA. Moreover, in the statistical analysis the results indicate that the *R*2-MOPSO is better with respect to the MOMBI-II and the SMS-EMOA. On the other hand, for DTLZ1 and DTLZ3 (which are shown in Tables 5 and 7, resp.), our approach clearly outperforms the SMS-EMOA and the MOMBI-II. For DTLZ1 the performance of SMS-EMOA decreased as we increased the number of objectives. The main problem of this algorithm is that the exact hypervolume calculation was replaced with an approximation, and when the number of objectives is raised it is necessary to increase the number of samples for the approximation as well. However, if the number of samples is increased, the computational cost would also increase. Moreover, in the DTLZ1 the numeric results are very similar for MOMBI-II and *R*2-MOPSO. However, as in DTLZ4 in the statistical analysis the *R*2-MOPSO is better than the MOMBI-II. Finally, for DTLZ3 our *R*2-MOPSO clearly outperformed the compared approaches. In this challenging problem, the SMS-EMOA was unable to converge.

## 5. Conclusions and Future Work

In this work, the incorporation of an indicator to guide the search of a PSO was proposed. Our proposed approach was validated using two- and three-objective function problems. The proposed *R*2-MOPSO obtained competitive results when compared to NSGA-II, MOEA/D, and SMS-EMOA using several test problems. Therefore, we can say that our approach is successful to work with multiobjective problems. However, since our main target was many-objective problems, we decided to study our approach with respect to this sort of problem. For this sake, we adopted scalable test functions and we compared our results with respect to a variation of a well-known hypervolume-based approach (SMS-EMOA) that approximates the hypervolume contributions, in order to have a more efficient performance. Our results indicate that our approach outperforms the SMS-EMOA with respect to the hypervolume. Finally, an important feature of this proposal is that it does not adopt Pareto dominance to guide the search; it is only adopted to report the found solutions.

As part of our future work, we would like to further investigate the use of the *R*2 indicator to restrict the size of external archives in other types of MOEAs.

## Figures and Tables

**Algorithm 1:**
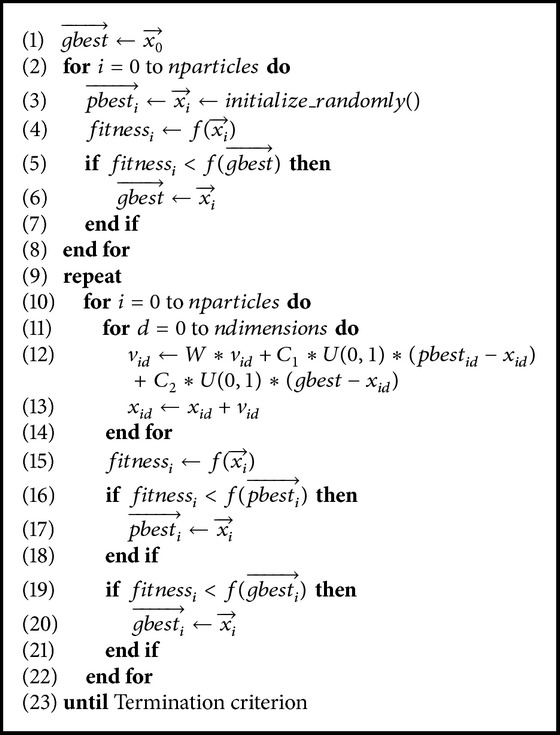
PSO algorithm.

**Algorithm 2:**
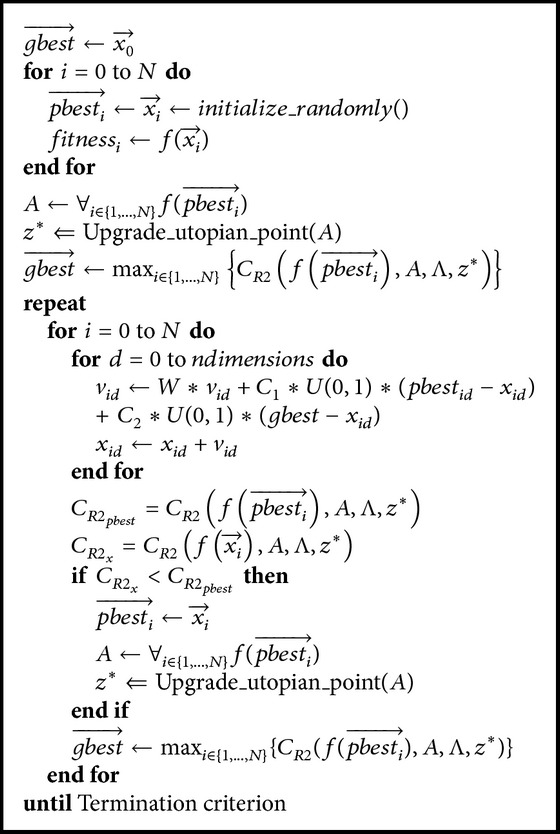
*R2*-MOPSO.

**Table 1:** Test problems adopted.

Test problem	# of variables	# of objectives
ZDT1–3, UF1–3	30	2
ZDT4, 6	10	2
DTLZ1	7	3
DTLZ2–4	12	3
UF8–10	30	3

**Table 2:** Adopted parameters for each MOEA.

NSGA-II	MOEA/D	SMS-EMOA	MOMBI-II	*R*2-MOPSO
*pc* = 1.0	*pc* = 1.0	*pc* = 1.0	*pc* = 1.0	*c*1 = 1.4
$pm=\frac{1}{n}$	$pm=\frac{1}{n}$	$pm=\frac{1}{n}$	$pm=\frac{1}{n}$	*c*2 = 1.4
*nc* = 15	*nc* = 15	*nc* = 15	*nc* = 30	*w* = 0.4
*nm* = 15	*nm* = 20	*nm* = 20	*nm* = 20	size_file = 100
	*T* = 20			

**Table 3:** Comparison of the results obtained by NSGA-II, MOEA/D, SMS-EMOA, MOMBI-II and *R*2-MOPSO with respect to the hypervolume with 20,000 evaluations for bi-objective problems and 30,000 for three objective problems.

Problem	NSGA-II	MOEA/D	SMS-EMOA	MOMBI-II	*R*2-MOPSO
ZDT1	120.644 ± 0.00	120.375 ± 0.40	120.643 ± 0.00	120.532 ± 0.03	120.455 ± 0.13
ZDT2	120.283 ± 0.01	119.019 ± 2.19	120.276 ± 0.03	119.001 ± 0.10	120.108 ± 0.16
ZDT3	128.752 ± 0.01	128.198 ± 1.21	128.749 ± 0.01	128.218 ± 0.18	128.453 ± 0.20
ZDT4	120.271 ± 0.55	118.496 ± 1.36	119.063 ± 1.49	119.127 ± 0.01	118.842 ± 1.02
ZDT6	116.090 ± 0.06	117.383 ± 0.03	115.938 ± 0.07	117.387 ± 0.12	116.281 ± 0.19
UF1	14.812 ± 0.01	14.789 ± 0.02	14.715 ± 0.01	14.943 ± 0.01	15.018 ± 0.02
UF2	5.359 ± 0.02	5.328 ± 0.02	5.343 ± 0.00	5.323 ± 0.02	5.437 ± 0.00
UF3	16.295 ± 0.01	16.248 ± 0.01	16.371 ± 0.02	16.705 ± 0.01	16.4009 ± 0.01
DTLZ1	0.177 ± 0.02	0.184 ± 0.00	0.188 ± 0.00	0.164 ± 0.01	0.168 ± 0.03
DTLZ2	0.690 ± 0.01	0.709 ± 0.00	0.740 ± 0.00	0.718 ± 0.01	0.721 ± 0.00
DTLZ3	0.961 ± 3.94	26.033 ± 1.14	2.032 ± 4.09	4.352 ± 3.21	7.229 ± 9.78
DTLZ4	0.646 ± 0.18	0.454 ± 0.22	0.637 ± 0.14	0.710 ± 0.12	0.721 ± 0.01
UF8	2.020 ± 0.01	2.021 ± 0.02	2.022 ± 0.01	2.016 ± 0.02	2.021 ± 0.00
UF9	1.435 ± 0.02	1.437 ± 0.01	1.435 ± 0.00	1.398 ± 0.01	1.438 ± 0.01
UF10	1.096 ± 0.01	1.099 ± 0.01	1.099 ± 0.02	1.1139 ± 0.03	1.100 ± 0.01

**Table 4:** HV: statistical analysis.

	ZDT1	ZDT2	ZDT3	ZDT4	ZDT6	UF1	UF2	UF3	DTLZ1	DTLZ2	DTLZ3	DTLZ4	UF8	UF9	UF10
*R*2-MOPSO/SMS-EMOA						+	+		+		+	+			
*R*2-MOPSO/MOMBI-II							+				+	+		+	−

**Table 5:** Comparison of the hypervolume's average obtained by MOMBI-II and *R*2-MOPSO for the DTLZ1 test problem with different number of objectives (*M*).

*M*	SMS-EMOA	MOMBI-II	*R*2-MOPSO
4	15.740759 ( )	15.96795 ( )	15.916804
5	23.495387 (+)	31.678247 ( )	31.647112
6	15.939020 (+)	63.881022 ( )	63.871206
7	37.503944 (+)	126.443144 ( )	127.956645
8	58.822058 (+)	253.535178 (+)	255.534288
9	71.723936 (+)	509.121631 (+)	510.093877
10	388.922373 (+)	1020.781209 (+)	1022.891997

**Table 6:** Comparison of the hypervolume's average obtained by MOMBI-II and *R*2-MOPSO for the DTLZ2 test problem with different number of objectives (*M*).

*M*	SMS-EMOA	MOMBI-II	*R*2-MOPSO
4	15.521335 ( )	15.490132 ( )	15.485958
5	31.598023 ( )	31.599134 ( )	31.581132
6	63.648437 ( )	63.639010 ( )	63.638744
7	127.672459 ( )	127.680517 ( )	127.680623
8	255.683900 ( )	255.693671 ( )	255.707244
9	511.666530 ( )	511.665803 ( )	511.660240
10	1023.617417 ( )	1023.521349 ( )	1023.471587

**Table 7:** Comparison of the hypervolume's average obtained by MOMBI-II and *R*2-MOPSO for the DTLZ3 test problem with different number of objectives (*M*).

*M*	SMS-EMOA	MOMBI-II	*R*2-MOPSO
4	0.0 (+)	0.310916 (+)	0.529142
5	0.0 (+)	1.231819 (+)	1.389182
6	0.0 (+)	0.201825 ( )	0.201977
7	0.0 (+)	2.351874 (+)	2.803984
8	0.0 (+)	5.925318 (+)	6.112229
9	0.0 (+)	38.102465 (+)	39.989576
10	0.0 (+)	15.234512 (+)	16.484128

**Table 8:** Comparison of the hypervolume's average obtained by MOMBI-II and *R*2-MOPSO for the DTLZ4 test problem with different number of objectives (*M*).

*M*	SMS-EMOA	MOMBI-II	*R*2-MOPSO
4	15.110649 (+)	15.221809 (+)	15.480663
5	31.489328 (+)	31.531203 (+)	31.580678
6	63.465142 (+)	63.530765 (+)	63.599144
7	127.641483 (+)	127.642368 (+)	127.678039
8	255.711370 (+)	255.721893 ( )	255.734554
9	511.741231 (+)	511.761209 (+)	511.781234
10	1023.679123 (+)	1023.680125 (+)	1023.691262
